# Implementation of an online thermal imaging to study the effect of process parameters of roller compactor

**DOI:** 10.1007/s13346-018-0493-9

**Published:** 2018-02-13

**Authors:** Chalak S. Omar, Michael J. Hounslow, Agba D. Salman

**Affiliations:** Department of Chemical and Biological Engineering, University of Sheffield, Mappin Street, Sheffield, S1 3JD UK

**Keywords:** Roller compaction, Online thermal imaging, Process parameters, Lactose

## Abstract

During roller compaction, not only the properties of the primary powder affect the product quality but also the process parameters. Any change in the process parameters during roller compaction will result in changing the properties of the ribbon. In this study, the temperature of the ribbon during production was monitored online using a thermal camera. The information from the thermal camera was used to explain the differences in ribbon properties at varying process parameters. Lactose powder was used as a primary powder, and ribbons were produced at different process parameters. The surface temperature of the ribbon during production was found to increase with increasing both the gap between the two rollers and the roller speed. This was attributed to the screw feeder speed, which increased to feed additional powder as required to adjust to the change in process parameters. Increasing the roller gap resulted in wider ribbons and decreased the percentage of fines in the product, which was a signature of better powder distribution across the roller width. The results were also supported by the uniform temperature distribution recorded across the ribbon width. It was found that increasing the roller speed during roller compaction decreased the width of the ribbon while increasing the percentage of fines in the product. The feeder screw speed was found to have a similar effect as the roller gap.

## Introduction

Roller compaction is a dry granulation technology in which high pressure is used to compress the primary powder particles into ribbons, which are then milled to granules of different size classes. The roller compaction process consists of three units working simultaneously: feeding, compaction and milling unit. The feeding unit provides and transports the powder to the compaction unit using different methods (gravity or screw feeder system). The compaction unit consists of two counter-rotating rollers which apply high stress on the powder [1]. The ribbon is then used to produce granules with the desired size after passing through a milling step.

During the roller compaction, not only the primary powder properties affect the product properties but also the process parameter during the ribbon production. There are few process parameters in the roller compactor which could be changed during production of compacts and granules. The compaction pressure, feeder screw speed, roller speed and the gap between the two rollers are the process parameters which affects the properties of ribbon and granules.

Compaction pressure is the most significant parameter that affects ribbon properties, as the compaction pressure determines the stress acting on the powder during compression. Increasing the compaction pressure increases the strength and the density of ribbon and granules [2–8]. Roller speed, screw speed and the gap between the rollers were also found to affect the quality of ribbon produced in the roller compactor [5, 6, 9, 10]. Roller speed, screw speed and the gap between the rollers were also found to affect the quality of ribbon produced in the roller compactor [5, 6, 9, 10].

The effect of process parameters on product quality has been investigated in the literature using different types of lactose [5], different types of MCC [9] and maize [6]. Process parameters were represented by the compaction pressure and the speed of the horizontal and vertical screws, while the product quality was represented by the granule friability. Lower granules friability was suggested to represent better quality of the product. It was found that the best product quality (low friability) for all materials was achieved by operating the roller compactor at high compaction pressure. The effect of the horizontal screw speed was different for different powders; the best quality of the product was obtained using low horizontal screw speed for lactose [5] and high horizontal screw speed for both MCC and maize [6, 9]. This could be due to the difference in flow properties of the powders.

The speed of the roller, at constant feeder screw speed, affects the amount of powder between the rollers and also the dwell time of the powder in the compaction zone which may affect the strength of the ribbon. Increasing the roller speed, by keeping both horizontal and vertical screw speed constant, was found to decrease the force required to break the ribbon, consequently, decreasing ribbon tensile strength and density [3, 4, 10]. This was due to the fact that at constant feeder screw speed, an increase in the roller speed decreased the amount of powder in the compaction zone; therefore, ribbons were thinner, lesser in width and easier to break. However, operating the roller compactor at constant (roller speed/horizontal feed screw/vertical feed screw constant at 1:5:25) ratios was found to have no effect on ribbon thickness, width, strength and density [10]. This was because the amount of powder between the rollers was always the same; therefore, ribbons with similar thickness and width were produced. An increase in the roller speed was also found to decrease the nip angle of MCC [11, 12] and Di-calcium phosphate dihydrate (DCPD) [13].

The gap between the two rollers is one of the process parameters which can be changed during the roller compaction. In the roller compactor, one of the rollers is movable while the other is stationary; the movable roller helps to fix the gap between the rollers. The gap determines the thickness of the produced ribbon, which may affect ribbon strength and porosity. Bindhumadhavan et al. [14] attempted to investigate the effect of the gap between the rollers on the behaviour of microcrystalline cellulose undergoing roller compaction using a gravity feeding system. They examined the effect of the roller gap on the maximum pressure applied to the powder during compaction. The pressure applied to the powder during roller compaction was found to decrease with increasing the gap between the two rollers. This could be due to the fact that increasing the gap between the rollers increases the amount of the powder in the gap region and reduces the applied stress on the powder (distributing the force acting on the powder over a wider thickness).

Miguélez-Morán et al. [15] investigated the effect of the gap between the rollers on the ribbon relative density. Microcrystalline cellulose MCC was used as a model powder, and ribbons were produced at two different gap settings. It was found that the average relative density of the ribbon was increasing with decreasing the gap between the rollers. This was due to the higher stress which was applied to the powder at lower roller gap; this is in agreement with the finding of Bindhumadhavan et al. [14].

During the roller compaction, the powder undergoes friction, deformation and fragmentation which then result in an increase in the product temperature. The product temperature was reported to increase with increasing the pressure during the roller compaction [2, 7, 16]. In these studies, a thermal camera was used to record the temperature of ribbon of different materials produced at different pressure. It was stated that different material exhibited varying temperature due to the differences in the behaviour of powders undergoing roller compaction. The higher temperature of ribbon at elevated pressure was attributed to the higher degree of deformation, fragmentation and friction between the particles. The online thermal imaging of ribbon was shown to be a useful technique to investigate the powder behaviour in the roller compaction [7]. The fact that the thermal camera can be used as an online technique is useful in monitoring the behaviour of the powder while roller compacted in real time. This means that there is a potential of using the feedback from this technique to control the product quality in real time by altering the roller compaction process parameters.

Operating the roller compactor at different process parameters affects both the product properties and the production rate. Therefore, it is essential to investigate the effect of the process parameters during the roller compaction process. In the current work, a detailed study will be performed to investigate the effect of the roller compaction process parameters on the product properties using lactose as a model powder. An online thermal imaging will be implemented to further understand the behaviour of the powder during the roller compaction at different process parameters.

## Material and methods

### Material

**Fig. 1:**
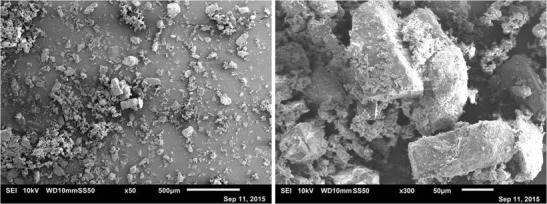
Scanning electron microscope images of powder: left ×50 magnifications and right ×300

α-Lactose monohydrate (200 M), was used as a model powder in this study. The powder was supplied by DFE Pharma, Germany. The particle size distribution of the primary powder was measured using Camsizer XT (Retsch Technology GmbH, Germany) which operates based on image analysis. The equipment uses compressed air to disperse the small and cohesive particles which ensures a record of the properties of a single particle. The particle size distribution was as follows: d_10_ = 12.5 μm, d_50_ = 47.5 μm and d_90_ = 118 μm. Scanning electron microscopy JEOL JSM-6010LA was used to obtain electron micrographs of the primary powder as shown in Fig. 1. It can be seen that the particles of α-lactose monohydrate (200 M) have a tomahawk-like shape.

The lactose powder used in this study were equilibrated prior to compaction at 25 °C and 20% RH for 3 days using a Binder KMF 240 climatic chamber (Binder, Germany) [8]. The equilibration of powder in the climatic chamber is faster than in a desiccator, due to the ability to control the air flow and temperature in the climatic chamber. Powders were spread in a thin layer in a plastic tray to increase the surface area of the powder.

### Methods

An Alexanderwerk WP120 (Alexanderwerk, Germany) roller compactor was used for the compaction of the lactose powder in this study. The WP120 has three systems working simultaneously: the feeding system, compaction system and milling system. The feeding system consists of a hopper for the powder and a horizontal screw feeder, which transports the powder from the hopper to the compaction system. Before the compaction system, on the screw feeder, there is a de-aeration system which takes the air out of the powder and improves powder flowability. The compaction system consists of two counter-rotating rollers of 120 mm in diameter and 40 mm width with knurled surfaces. The two rollers apply high pressure on the powder using a hydraulic system, which gives a hydraulic pressure in the range of 18–230 bar. The time of compression can be controlled by changing the speed of the rollers from 3 to 13 rpm. The thickness of the ribbon can be defined by the gap between the two rollers which can be changed between 1 and 4 mm. There are two cheek plates on both sides of the rollers used to reduce the leakage of the powder during compression.

To eliminate the effect of humidity in the laboratory on the powder relative humidity, a GenRH-A (London, UK) humidity generator was connected to the top of the hopper on the roller compactor. The humidity generator was operated at the same conditions as the climatic chamber; this ensured a constant humidity condition of the powder during roller compaction same as that used for the storage of the powder prior to compaction.

**Fig. 2:**
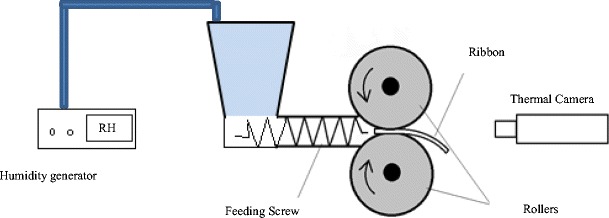
Schematic diagram of the online thermal imaging and the humidity generator setup

The ribbon surface temperature was recorded during production using a FLIR A655sc thermal camera (FLIR-Sweden). The online thermal imaging setup is shown in Fig. 2, where the camera was facing the two rollers and recording the temperature of the exiting ribbon directly. Ribbon temperature was recorded once the gap and pressure in the roller compactor had reached steady state. Each experiment was recorded for 60 s at a frame rate of 50 fps. Images from each experiment were then analysed using FLIR R&D software to find the maximum temperature in each frame. Then, the average maximum temperatures for all frames were determined.

During the roller compaction, an amount of fine (un-compacted powder) are produced, this is a main disadvantage of the roller compaction process. The amount of fines was defined as the amount of un-compacted powder (particles which are smaller than the d_90_ of the original powder) which is produced along with the ribbon. To determine the amount of fines, the entire product (ribbon and fines) was collected for a set period of time during the operation of the roller compactor. Then, the ribbons were separated from the fines by sieving (Retsch GmbH, Germany) at 0.2 mm amplitude for 1 min. From the total mass of the product and the mass of fines, the weight percentage of fines was determined.

The width of the ribbons was measured using a digital calliper. A total of ten ribbons were selected randomly, and the width was measured for each of them. The average of the ten values was then determined at each condition.

A Zwick/Roell Z 0.5 (Zwick/Roell, Germany) test machine was used to find the maximum force required to break the ribbon using the three-point bend test. The setup consisted of two supports to hold the ribbon and a load which was applied to the centre of the ribbon at a speed of 0.1 mm/min. The maximum force was then recorded and used to determine the tensile strength of the ribbon using Eq. (1) [2, 17–21]

1 $$ {\sigma}_t=\frac{3{F}_{max}L}{2b{d}^2} $$

where *σ*_*t*_ is ribbon tensile strength (N/mm^2^), *F*_max_ is the maximum force required to break the ribbon (N), *L* is the distance between the two supports (mm), which was calculated depending on ribbon thickness using the formula *L* = (16 ± 1)*d* [17, 18], *b* is width of the ribbon (mm) and *d* is the thickness of ribbon (mm).

X-ray images of ribbons produced from the roller compaction were obtained using micro-CT 35 (Scanco Medical AG, Switzerland). Samples of ribbon of lactose were attached to a sample holder. The X-ray beam was operated at a voltage of 45 kV, a current of 177 μA and a power of 8 W. The voxel size used was 3.5 μm.

Images from the X-ray machine were then analysed using Image J software to determine the porosity of the ribbon. The black pixels in the X-ray images indicate the air (pores), and the white pixels indicate the powder. The number of black pixels (air) was determined and divided by the total number of pixels (air and powder); this ratio represents the porosity of the ribbon.

## Results and discussion

### Effect of the roller speed

The speed of the roller controls the dwell time of the powder between the two rollers and also the amount of powder between the rollers which may affect the strength of the ribbon. In order to examine the effect of the roller speed, the powder was compacted at a constant pressure of 50 bar and using different speeds of the roller (3, 4, 5 and 6) rpm. The gap between the rollers was kept constant at 3 mm by using the automatic feedback system which alters the speed of the feeder screw to maintain a constant gap.

**Fig. 3:**
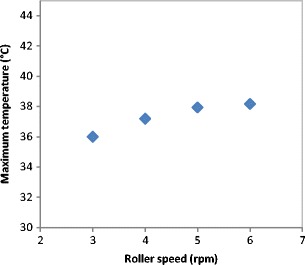
The surface temperature of the ribbon during the production at different roller speed

The surface temperature of the ribbon was determined using an online thermal camera during the roller compaction. Figure 3 shows the maximum temperature of the ribbon during the production at a different roller speed. It can be seen that the temperature of the ribbon increased slightly with increasing the speed of the roller during production. The slight increase in the ribbon temperature with increasing the roller speed could be attributed to the increase in the internal friction of the powder as a result of increasing the speed of the powder while passing between the rollers. Another reason for the increase in the temperature could be the increase in the feeder screw speed (see Table 1) while attempting to maintain constant gap during ribbon production. This will increase the internal friction between the particles and increase the temperature of the ribbon. This is in agreement with the finding of Osborne [22] in that the temperature of ribbon of maltodextrin was found to increase slightly with increasing the roller speed in the range of (3–9 rpm).

**Table 1:** Feeder screw speed at different roller speed

Roller speed (rpm)	Feeder screw speed (rpm)
3	59.5
4	69
5	76
6	81.3

**Fig. 4:**
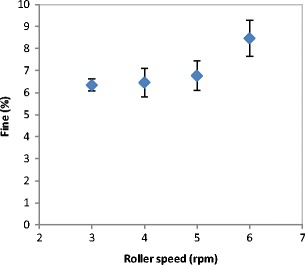
Fine percentage produced during the production of ribbon at different roller speed

The percentage of fines produced during the roller compaction at different roller speed was used to evaluate the product quality. Figure 4 shows the percentage of fines produced during compaction at different roller speed. The fine percentage was found to increase with increasing the speed of the roller during the roller compaction. The increase in the roller speed during the compaction increases the speed of the powder and decreases the time which the powder spends in the compaction zone. The higher the speed of the roller the lesser the time for powder in the compaction zone. This means that the powder will not have enough time to distribute evenly across the width of the roller. This will result in less powder availability at the edges which leads to a decrease in the stress applied to the powder at the edges consequently poor binding and a larger amount of fines at higher roller speed.

**Fig. 5:**
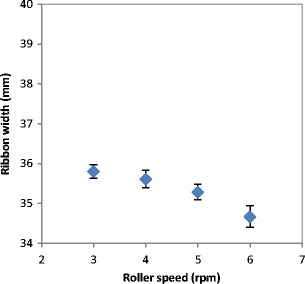
The width of ribbon produced from the three types of lactose at different roller speed

The poor distribution of the powder across the width of the roller at higher roller speed resulted in less powder being present at the edges which affected the width of the produced ribbon. Figure 5 shows the width of ribbon produced from lactose at different roller speed. It can be seen that the width of the ribbon is decreasing with increasing the speed of the roller during the production. Increasing the speed of the roller during the compaction decreases the amount of the powder at the edges. This will result in the larger amount of fines in the product (see Fig. 4) and consequently in ribbons with a smaller width. This result is in agreement with the finding of Miguélez-Morán et al. [15], who reported that wider ribbons can be produced when lower roller speed used during the production of ribbon using MCC avicel PH102.

**Fig. 6:**
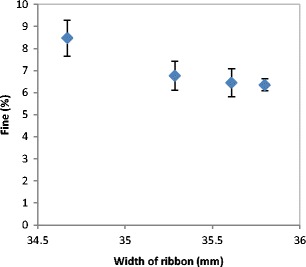
The relationship between the fine percentage and the width of the ribbon

From Figs. 4 and 5, it can be concluded that the percentage of fines produced during the roller compaction is related to the width of the ribbon as shown in Fig. 6. It was found that the higher the width of the ribbon the lesser the percentage of fines in the product.

**Fig. 7:**
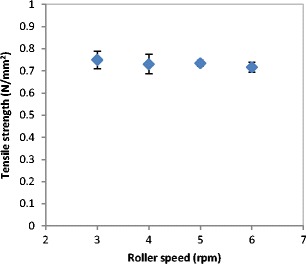
The tensile strength of ribbon produced at different roller speed

The increase in the roller speed was found to have an insignificant effect on the ribbon tensile strength Fig. 7. It can be seen that the ribbon tensile strength decreased very slightly with increasing the speed of the rollers during production. The slight and insignificant decrease in the ribbon strength could be attributed to the decrease in the dwell time (the time which the powder spends under compression) with increasing the roller speed.

**Fig. 8:**
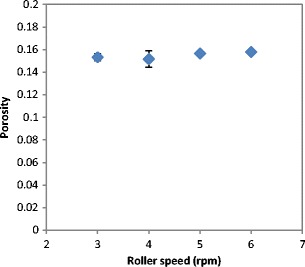
The porosity of ribbon produced at different roller speed

The porosity of ribbon was determined using the X-ray tomography. The porosity of ribbon was determined and plotted against the roller speed in Fig. 8. It can be seen that the increase in the roller speed had no effect on the ribbon porosity. This result is supported by the finding in Fig. 7 where roller speed was found to have an insignificant effect on the ribbon tensile strength. The minor effect of the roller speed on the ribbon tensile strength and porosity is in agreement with the findings in the literature [10, 22]. Osborne [22] reported that the increase in the roller speed during compaction has a minor effect on the tensile strength of ribbon produced from maltodextrin, microcrystalline cellulose (MCC) and sodium chloride. In the current work, the gap between the rollers was controlled using the automatic feedback system which adjusts the feeder screw speed. This means that the increase in the roller speed increases the feeder screw speed in order to feed more powder and maintain the gap. The constant gap between the rollers with increasing the roller speed resulted in ribbons with similar strength and porosity. This finding is interesting because it is suggesting that the production rate of ribbon of lactose powder could be increased by increasing the speed of the roller knowing that this will not affect the product strength and porosity.

### Effect of the gap between the rollers

The gap between the two rollers is one of the process parameters which can be changed during the roller compaction. Increasing the gap during the production of ribbon increases the amount of powder between the rollers and results in thicker ribbon. The increase or decrease in the amount of powder between the two rollers may affect the stress applied to the powder at specific roller force. Previous work has been carried out to investigate the effect of the roller gap on the nip angle and the maximum pressure applied to the powder [14, 23]. It was found that the nip angle increased with increasing the roller gap during the roller compaction of MCC [23]. This may affect the properties of the produced ribbon.

In this section, the effect of varying the roller gap on ribbon properties was studied using lactose. The roller compactor was operated at a fixed hydraulic pressure of 50 bar, the roller speed was set to 3 rpm and the gap between the rollers were (1.5, 2, 3 and 4) mm. To control the set gap, the feeder screw speed was varying automatically using the feedback system which adjusts the feeder speed to maintain the set gap.

**Fig. 9:**
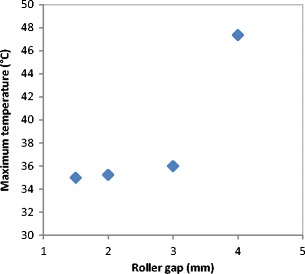
The maximum temperature of the ribbon during the production at different roller gap

**Fig. 10:**
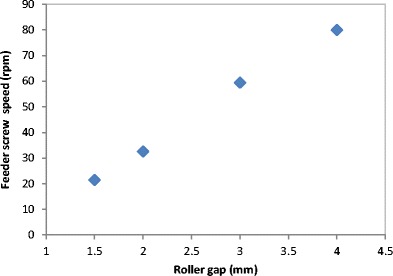
Feeder screw speed at different roller gap

Figure 9 shows the maximum temperature of the ribbon during the production at different roller gap setting. It can be seen that the temperature of the ribbon during production increased with increasing the gap between the two rollers. The increase in the roller gap increases the speed of the feeder screw (see Fig. 10) in order to deliver more material and fill the gap between the rollers. This is achieved by operating the roller compactor with the feedback system turned on, which adjusts the feeder screw speed according to the gap setting. The increase in the feeder screw speed, with increasing the roller gap, increases the internal friction between the particles and between the barrel wall and particles which increase the powder temperature. This was the reason for the increase in the ribbon surface temperature with increasing the roller gap. Another reason could be the higher heat accumulation in thicker ribbon which is produced at larger gap; a thicker ribbon loses heat at a slower rate in comparison to a thinner ribbon.

**Fig. 11:**
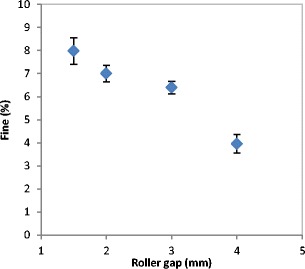
The percentage of fines produced with the ribbon during the production at different roller gap

**Fig. 12:**
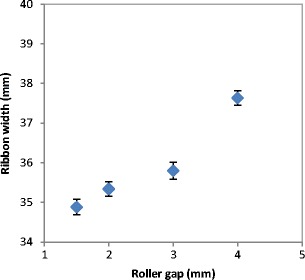
The width of ribbon produced from the three types of lactose at different roller gap

The quality of the product at different roller gap settings was also evaluated by determining the fine percentage in the product. As mentioned earlier, the fine is one of the main disadvantages of the roller compaction; therefore, decreasing the fines in the product increases the overall efficiency of the process. The fine percentage during the roller compaction of lactose was determined and plotted against the roller gap as shown in Fig. 11. It can be seen that the increase in the roller gap during the compaction resulted in a significant decrease of the fine percentage in the product. This is supported by the finding in Fig. 12, which shows the width of the ribbon produced at varying roller gap. It can be seen in Fig. 12 that the width of the ribbon is increasing significantly with the increase in the roller gap during the production of ribbon. This confirms the result in Fig. 11, as the fine is usually coming from the edges of the ribbon, therefore, an increase in the width of the ribbon gives an indication of a lower amount of fines in the product.

The lower percentage of fines and the wider ribbon produced at high roller gap is due to a better powder distribution across the width of the roller. Increasing the gap size between the two rollers means more space for the powder to spread to the edges of the rollers. The better distribution of the power to the edges at larger roller gap could improve the stress distribution across the width of the roller. The stress across the roller width is more uniform when larger gap size is used during the compaction. This results in a wider ribbon and therefore a lower percentage of fines in the product.

**Fig. 13:**
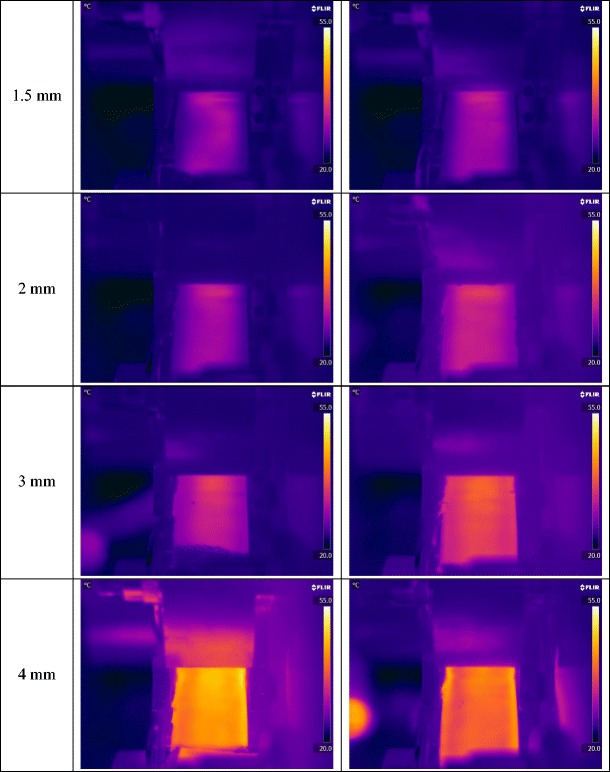
Thermal images of the ribbon during production at different roller gag

**Fig. 14:**
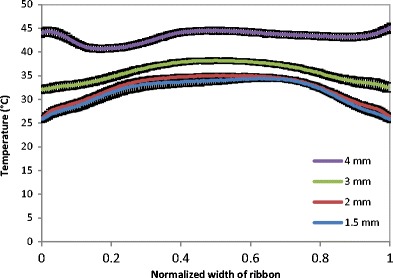
Temperature distribution across the width of ribbon produced at different roller gap

The better distribution of the powder at larger gap size was investigated further with the online thermal imaging camera. Figure 13 shows a series of thermal images of the ribbon during production at different roller gap. It can be seen that the temperature of ribbon increased with increasing the gap between the rollers, the brighter the colour in the thermal images the higher the temperature. It can also be seen that the increase in the roller gap during ribbon production resulted in a better uniformity of temperature distribution across the ribbon width. The thermal images were further analysed to determine the temperature distribution across the width of the ribbon (see Fig. 14). It can be seen that the production of ribbon at small gap (1.5 mm) resulted in high temperature at the centre and lower at the edges. The high temperature at the centre of the ribbon is an indication of higher stress which was applied as a result of a higher amount of powder at the roll centre, and then low temperature indicates the low stress at the edges. This resulted in weak ribbon at the edges and therefore in ribbons with less width (Fig. 12) and higher fine percentage (Fig. 11). Increasing the gap between the rollers gives more space for the powder to distribute evenly across the width of the roller. This resulted in a uniform temperature distribution across the width of the ribbon (see Figs. 13 and 14), which is believed to be due to the uniform stress applied by the roller across its width. This was the reason of wider ribbon with a lesser percentage of fines at larger roller gap (Figs. 11 and 12).

**Fig. 15:**
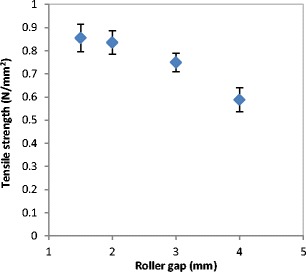
The tensile strength of ribbon at different roller gap

**Fig. 16:**
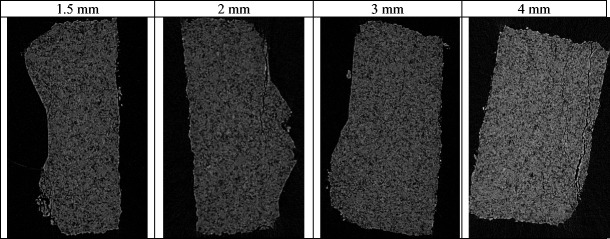
X-ray images of ribbon produced at different roller gap

**Fig. 17:**
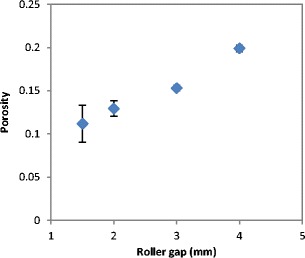
The porosity of ribbon produced at different roller gap

The tensile strength of ribbons produced at different roller gap is shown in Fig. 15. Figure 16 shows X-ray images of ribbons produced at different gap which was used to determine the porosity of ribbon as shown in Fig. 17. The tensile strength of ribbons decreased, and the porosity increased with increasing the roller gap. The decrease in the tensile strength of ribbon is attributed to the fact that increasing the roller gap increases the amount of the powder between the two rollers. The higher amount of the powder at the gap between the rollers is believed to decrease the stress applied on the powder between the two rollers during the compaction. The decrease in the maximum stress applied on the powder with increasing the roller gap was reported previously by [14, 23, 24]. This was attributed to the fact that increasing the roller gap increases the amount of the powder between the rollers which resulted in distributing the force acting on the powder over a wider thickness, therefore, decreasing the stress. The decrease in the stress applied on the powder during the roller compaction, as a result of increasing the roller gap, resulted in ribbons with lower tensile strength (see Fig. 15) and higher porosity (see Fig. 17). This is in agreement with the finding of [14, 22, 23] in that increasing the roller gap during the compaction decreases both the strength and the density of ribbon of MCC and sodium chloride.

### Effect of the feeder screw speed

In roller compaction, a screw feeder is, sometimes, used to transport the powder from the hopper to the compaction zone. A change in the feeder screw speed (at constant pressure and roller speed) results in changing the gap between the rollers. In the previous sections, the gap between the rollers was controlled using the automatic feedback system which adjusts the feeder screw speed to maintain the gap constant; therefore, the screw speed will change automatically. In order to investigate the effect of the feeder screw speed, the powder was compacted using a fixed hydraulic pressure of 50 bar, a roller speed of 3 rpm and screw feeder speed of 20, 40, 60 and 80 rpm.

**Fig. 18:**
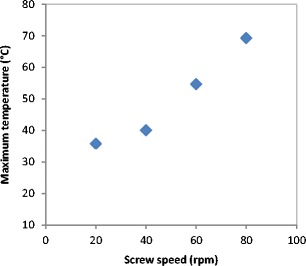
The surface temperature of ribbon produced at different feeder screw speed

An increase in the feeder screw speed results in an increase in the amount of powder in the compaction zone and then increases the gap between the rollers (at fixed roller speed). Therefore, it is expected that the feeder screw will have the same effect as the roller gap. Figure 18 shows the surface temperature of the ribbon during the production at different feeder screw speed. The temperature of ribbon increased with increasing the feeder screw speed. This was because of the increase in the powder internal friction as a result of the increase in the screw speed. The Same result was found when increasing the roller gap since the increase in the gap increases the feeder screw speed due to the use of the automatic feedback system.

**Fig. 19:**
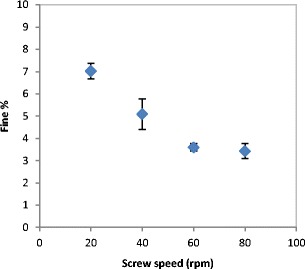
Percentage of fines produced at different feeder screw speed

**Fig. 20:**
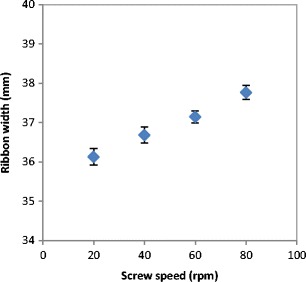
The width of ribbon produced at different feeder screw speed

Figures 19 and 20 show the fine percentage and the width of ribbon produced at different feeder screw speed. It can be seen that the fine percentage decreased and the width increased with increasing the speed of the feeder screw. As mentioned earlier, the increase in the feeder screw speed increases the roller gap.

At a larger gap, the powder will have more space to distribute evenly across the width of the roller. The uniform distribution of the powder across the width of the roller is believed to result in a uniform stress being applied to the powder across the roller width. This will result in ribbons with a higher width (see Fig. 20) and reduce the percentage of the fines in the product (see Fig. 19).

**Fig. 21:**
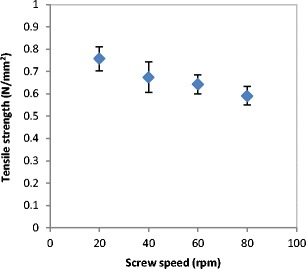
The tensile strength of ribbon produced at different feeder screw speed

**Fig. 22:**
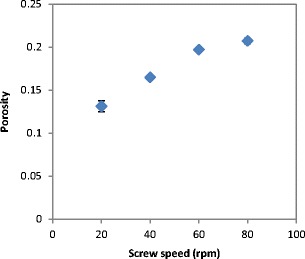
The porosity of ribbon produced at different feeder screw speed

The tensile strength and the porosity of ribbon produced at different feeder screw speed are shown in Figs. 21 and 22 respectively. The tensile strength decreased, and the porosity increased with increasing the feeder screw speed. The result in Figs. 21 and 22 are similar to the finding in Figs. 15 and 17 respectively since the increase in the roller gap has the same effect as increasing the feeder screw speed. The decrease in the tensile strength and the increase in porosity of ribbon with increasing the feeder screw speed are due to the higher amount of powder between the rollers at higher screw speed. The presence of a large amount of powder between the rollers decreases the stress applied to the powder. This will then decrease the ribbon tensile strength and increase the porosity of ribbon.

## Conclusions

The effect of the process parameters of the roller compaction was investigated. An online thermal imaging was employed to determine the maximum temperature of the ribbon during production at various process variables. The amount of fines, width, strength and porosity of ribbon were used to evaluate the product quality. It was found that increasing the roller speed during the roller compaction decreases the width of the ribbon, which consequently increases the fine percentage in the product. However, the speed of the rollers was found to have a minor effect on the tensile strength and porosity of the ribbon. This is an attractive finding, as it proposes the possibility of increasing the production rate of ribbon of lactose powders with increasing the roller speed, knowing that this will not affect the ribbon strength and porosity.

The gap between the two rollers and the screw feeder speed was also found to affect the powder compaction behaviour and ribbon properties. The temperature of the ribbon during production was found to increase with increasing both the gap size between the rollers and the screw feeder speed. An increase in the roller gap means more space for the powder to move and distribute across the width of the rollers. The better distribution of the powder across the width of the roller at larger gap size resulted in a uniform temperature distribution and, consequently, in wider ribbons and a lower percentage of fines. The tensile strength of ribbon decreased, and the porosity increased with increasing the roller gap and screw speed.
